# Early Clinical Experience with a Polymer-Free Biolimus A9 Drug-Coated Stent in DES-Type Patients Who Are Poor Candidates for Prolonged Dual Anti-Platelet Therapy

**DOI:** 10.1371/journal.pone.0157812

**Published:** 2016-06-30

**Authors:** Tim Kinnaird, Mehmood Butt, Fairoz Abdul, Khaled Yazji, Ahmed Hailan, Sean Gallagher, Nicholas Ossei-Gerning, Alexander Chase, Anirban Choudhury, David Smith, Richard Anderson

**Affiliations:** 1 Department of Cardiology, University Hospital of Wales, Cardiff, United Kingdom; 2 Department of Cardiology Morriston Cardiac Centre, Swansea, United Kingdom; Harvard Medical School, UNITED STATES

## Abstract

**Introduction:**

Prolonged dual anti-platelet therapy (DAPT) may cause excess bleeding in certain patients. The biolimus-A9 drug-coated stent (BA9-DCS) has a rapid drug-elution profile allowing shortened DAPT. Data were gathered on the early experience implanting this stent in drug-eluting stent eligible patients deemed to be at high risk of bleeding.

**Background and Methods:**

The demographics, procedural data and clinical outcomes were gathered prospectively for 249 patients treated with a BA9-DCS stent at 2 UK centres, and compared to a cohort of patients treated in the same period with drug-eluting stents (PCI-DES).

**Results:**

Operator-defined BA9-DCS indications included warfarin therapy, age, and anaemia. Patients receiving a BA9-DCS were older (71.6±11.8 vs. 64.8±11.6yrs, p<0.001), more often female (38.2 vs. 26.8%, P<0.001), and more likely to have comorbidity including chronic kidney disease or poor LV function than PCI-DES patients. The baseline Mehran bleed risk score was also significantly higher in the BA9-DCS group (19.4±8.7 vs. 13.1±5.8, p<0.001). Of the BA9-DCS cohort, 95.5% of patients demonstrated disease fitting NICE criteria for DES placement. The number of lesions treated (1.81±1.1 vs. 1.58±0.92, p = 0.003), total lesion length (32.1±21.7 vs. 26.1±17.6mm, p<0.001), number of stents used (1.93±1.11 vs. 1.65±1.4, p = 0.007) and total stent length (37.5±20.8 vs. 32.4±20.3, p<0.01) were greater for BA9-DCS patients. DAPT was prescribed for 3.3±3.9 months for BA9-DCS patients and 11.3±2.4 months for PCI-DES patients (p<0.001). At follow up of 392±124 days despite the abbreviated DAPT course stent related event were infrequent with ischemia-driven restenosis PCI (2.8 vs. 3.4%, p = 0.838), and stent thrombosis (1.6 vs. 2.1%, p = 0.265) rates similar between the BA9-DCS ad PCI-DES groups. After propensity scoring all clinical end-points were similar between both cohorts.

**Conclusions:**

This early experience using polymer-free BA9 drug-coated stents in drug-eluting stent type patients at risk of bleeding are encouraging. Further studies are warranted.

## Introduction

Prolonged dual anti-platelet therapy (DAPT) exposes patients to the risk of bleeding complications after PCI. In the CURE trial the absolute excess risk of major bleeding with clopidogrel over placebo at 12-months was 0.5–1.0% [1]. Additionally, in the recent DAPT trial, extending clopidogrel from 12 to 30 months increased the relative risk of major bleeding by 56% vs. placebo [2]. Furthermore, landmark analyses of the TRITON, PLATO and PRODIGY trials clearly demonstrate that the risk of bleeding with either clopidogrel vs. placebo or prasugrel/ticagrelor vs. clopidogrel increases over time, with early and continuing separation of the bleeding curves [3–5]. However, most trials of contemporary stents and/or antiplatelet agents exclude patients at risk of bleeding. These “non-comers” are in important group in real-world practice that often involves treating patients with baseline anaemia, a bleeding history/bleeding diathesis, those treated with warfarin, or those who cannot take prolonged DAPT courses (perhaps due to compliance issues or because of the need for early non-cardiac surgery) [6–7]. Therefore, in routine clinical practice the excess risk of bleeding with prolonged DAPT courses is likely to occur more frequently than observed in randomised clinical trials.

Previous registry data combined with observational data from clinical trials have demonstrated beyond doubt that bleeding after PCI is a significant event, with a close association with morbidity and mortality for at least 12-months after the procedure [8–9]. Several clinical trials of shortened courses of DAPT have been conducted recently suggesting that shortened courses of DAPT (3–6 months) do not appear to lead to excess ischaemia outcomes. A major limitation of these trials is a lack of power to detect infrequent events such as stent thrombosis. Indeed, in the recent large DAPT trial (in which patients were treated with first and second generation drug-eluting stents) prolonging DAPT duration to 30-months reduced the risk of stent thrombosis vs. standard therapy at a cost of increased bleeding. In assimilating many of the recent DAPT duration trials, the most recent ESC guidelines on post-PCI anti-platelet treatment recommend a 6-months course of DAPT after DES implantation for stable angina, 12-months acute coronary syndrome PCI, but as little as 3-months DAPT duration when patients are deemed to be at high risk of bleeding [10].

However, as well as shortened courses of DAPT after implantation of contemporary second-generation stents, newer stent technologies also provide further potential to maintain optimal ischaemia outcomes whilst avoiding bleeding associated with prolonged DAPT. The BioFreedom stent (Biosensors SA, Switzerland) is a stainless steel platform engineered to allow the biolimus A9 drug to be applied directly to its surface obviating the need for any polymer. As a result, the stent is polymer-free and demonstrates rapid drug elution profile allowing accelerated vessel healing. This has the potential to facilitate a shortened duration of DAPT without compromising TLR and MACE [11]. The recent LEADERS FREE trial demonstrated reduced target lesion revascularisation and myocardial infarction rates in patients treated with a BA9-DCS stent compared to patients treated with bare-metal stents [12]. The purpose of the current study was to gather clinical and PCI procedural data on our early experience implanting the BioFreedom drug-coated stent (BA9-DCS) in drug-eluting stent (DES) eligible patients deemed to be at high risk of bleeding with prolonged DAPT.

## Methods

The University Hospital of Wales, Cardiff and Morriston Cardiac Centre, Swansea provide tertiary cardiac care to a population of 2.3 million people and combined perform in excess of 2,500 PCIs/year. The BA9-DCS became available for clinical use in the United Kingdom in January 2014 and was implanted at both centres at the interventional cardiologists discretion. Treatment was targeted to patients in whom it was deemed undesirable to prescribe prolonged DAPT therapy. Baseline demographics, procedural data and outcomes were gathered and entered prospectively from 249 consecutive patients treated with a polymer-free biolimus A9 drug-coated stent over a 12-month period. The Cardiff and Vale University Health Board audit department approved the study. As all data acquisition and analysis was retrospective, and because patient records/information were anonymized and de-identified prior to analysis, patient consent was not sought. To contextualise the characteristics of the BA9-DCS cohort we gathered baseline, procedural and outcome data on all other patients undergoing PCI with drug-eluting stents (PCI-DES) at our institutions during the same time period. Consecutive patients with de-novo disease treated with the BioFreedom stent were included apart from those presenting in cardiogenic shock. Patient demographics and procedural data were retrieved from the national British Cardiovascular Intervention Society Database (BCIS) Central Cardiac Audit (CCAD) database. The standard CCAD definitions of comorbidity were used for the study purposes [13]. The indication for BA9-DCS use and recommended DAPT duration were recorded prospectively. Mortality was recorded from the Welsh Demographic Service database (a centrally held record of all deaths in Wales). To define DES eligibility, we applied the National Institute of Clinical Excellent (NICE) criteria i.e. any stent <3.0mm in diameter or >15mm in length. Post-procedure untreated lesions were those in which PCI was unsuccessful or not attempted.

For follow-up, the in-hospital causes of death were derived from internal database analysis. For deaths in the community the patient’s general practitioner was contacted for cause of death. Repeat procedure data were derived from an internal angiographic database and the CCAD database. Stent thrombosis (ST) was defined as definite, probable or possible as per the ARC criteria. For completeness, patients’ details were crosschecked with all interventional centres in Wales to ensure TLR or stent thrombosis had not been treated in another centre. Ischemia-driven TLR (ID-TLR) was defined as repeat PCI to the study stent or the segment 5mm either proximally or distally that was performed because of a recurrence of chest pain or presentation with an acute coronary syndrome. Myocardial infarction (MI) was defined using the universal definition of myocardial infarction criteria. Bleeding was defined using BARC criteria. Major adverse cardiac outcomes were the combination of death, ST, and ID-TLR with episodes treated in a hierarchical fashion ie death was the most important followed by stent thrombosis and then target lesion revascularisation. Data were gathered for audit purposes to ensure patient safety and permission for this granted by the hospital audit department. Clinical outcome data were collected by research registrars (FA, KY) who were not directly involved in the patients’ care.

Propensity score analysis was performed in order to balance for covariates that might bias estimates for causal inferences. The variables that were controlled were age, sex, weight, presentation, previous MI, angina status, diabetes status, history of renal insufficiency, congestive heart failure, Q-wave on ECG, hypertension, CVA and PVD. A propensity score for each patient was obtained from a logistic regression using Inverse Probability of Treatment Weights. The adjusted event rates (adjusted Kaplan-Meier estimates) and the adjusted hazard ratios and p-value were reported. Continuous data were expressed as mean (SD) and comparison between groups was performed using Student T-Test. Categorical data are presented as frequencies and percentages, and were compared using a chi-square statistics or the Fisher’s exact test where indicated.

## Results

### Baseline Demographics

**Fig 1:**
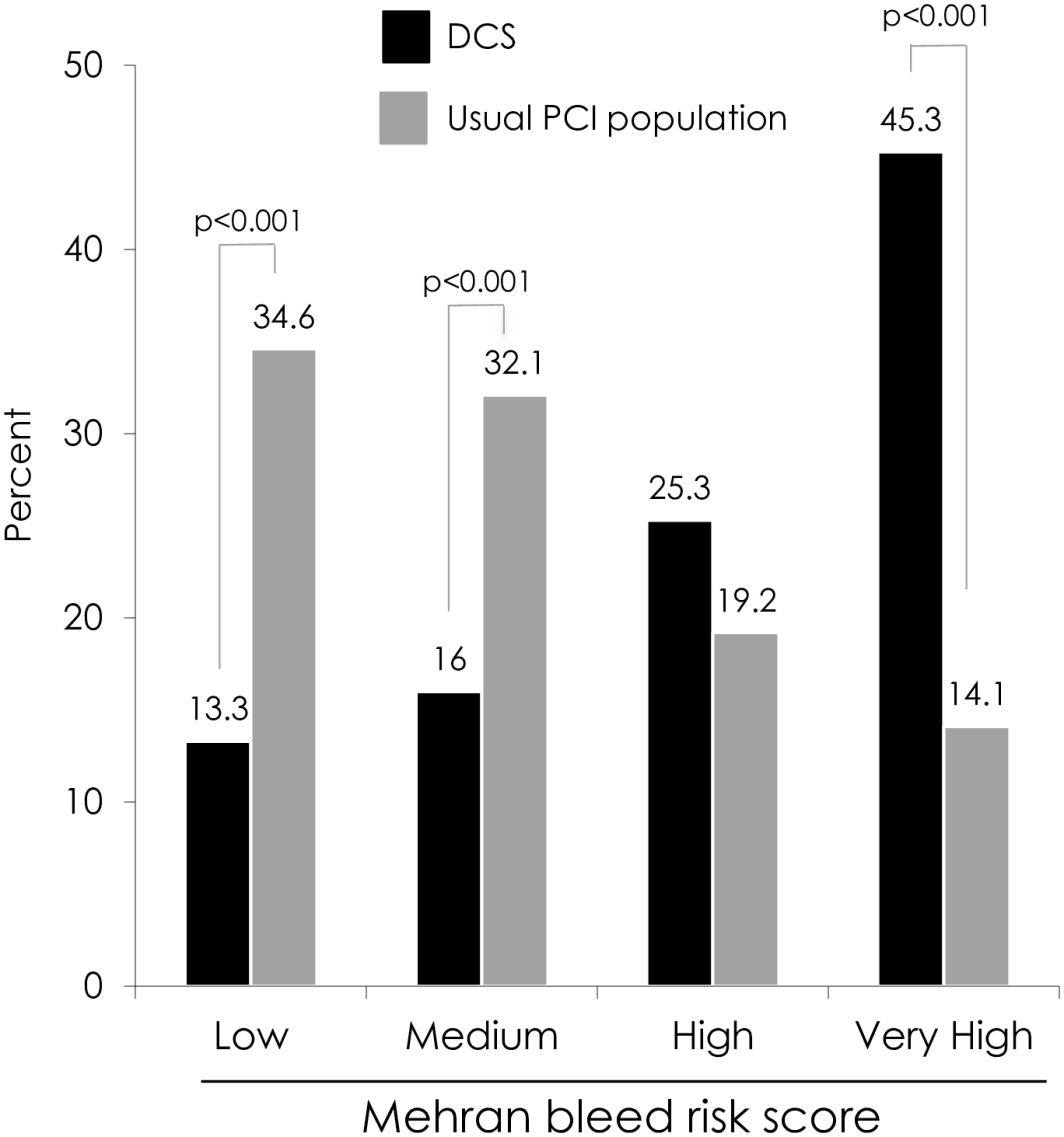
Mehran bleed risk subgroups patient groups.

A total of 249 patients underwent stenting with a BA9-DCS between January 2014 and February 2015 and as such represents 8.8% of the total volume of PCI procedures undertaken at the 2 centres in that time period. The baseline demographics of the BA9-DCS and PCI-DES patients are listed in Table 1. BA9-DCS patients were older (71.6±11.8 vs. 64.8±11.6 years, p<0.001), more often female (38.2 vs. 26.8%, p<0.001), lower body weight (77.8±18.0 vs. 82.9±17.2kg, p<0.001), and more likely to present with an ACS (83.1 vs. 73.6%, p<0.001) than PCI-DES patients. Several other characteristics associated with adverse outcomes including history of hypertension, baseline anaemia, raised creatinine, severe LV dysfunction, and the presence of a Q wave on the ECG were also more frequent in the BA9-DCS cohort. The recorded primary indication for BA9-DCS use was driven by concerns regarding prolonged DAPT with the indications including concurrent warfarin therapy (27.5%), the need for early non-cardiac surgery (25.1%), possible non-compliance with DAPT (20.9%), baseline anaemia/history of bleeding (12.6%), advanced age (operator defined, 7.2%) and history of active cancer (6.6%). Patients in BA9-DCS cohort had a mean of 1.4 indications for shortened DAPT. As might be expected oral anti-coagulation use was more prevalent in the BA9-DCS cohort (25.1 vs. 5.8%, p<0.0001). Additionally, the mean Mehran score for the BA9-DCS cohort was 19.4±8.7 compared to 13.1±5.8 in the PCI-DES cohort (p<0.0001). Fig 1 illustrates the Mehran bleed risk category by treatment group, indicating an excess of BA9-DCS patients in the highest risk group (45.3 vs. 14.1%, P<0.0001) compared to PCI-DES patients.

**Table 1:** Baseline demographics of patients treated with a BA9 drug-coated stent vs. PCI-DES population.

	DCS Cohort (n = 249)	PCI-DES cohort (n = 1630)	p value
Age (±SD)	71.6±11.8	64.8±11.6	<0.001
Age≥75years (%)	46.2	20.7	<0.001
Female (%)	38.2	26.8	<0.001
ACS presentation (%)	83.1	73.6	<0.001
Diabetes (%)	23.7	22.4	0.615
Weight (kg)	77.8±18.0	82.9±17.2	<0.001
Hypertension (%)	74.0	65.1	<0.001
Smoking history (%)	67.7	65.2	0.416
Peripheral vascular disease (%)	2.6	3.0	0.861
Cerebrovascular accident (%)	1.5	1.9	0.823
Chronic kidney disease (%)	5.7	2.5	<0.001
Prior MI (%)	35.2	19.2	<0.001
Prior CABG (%)	6.8	5.9	0.529
Prior PCI (%)	15.7	24.4	<0.001
Ejection fraction <30% (%)	16.1	8.2	<0.001
Q wave present (%)	15.3	7.1	<0.001
Mehran bleed score	19.4±8.7	13.1±5.8	<0.001
Baseline haemoglobin (g/l)	124.2±21.2	141.0±16.9	<0.001
Baseline white cell count (10^9^/l)	9.4±5.1	9.2±2.8	0.414
Baseline creatinine (umol/l)	116.6±97.1	85.9±28.9	<0.01
Oral anticoagulation (%)	25.1	5.8	<0.001

### Procedural Data

Reflective of increased age and a greater frequency of female patients, the BA9-DCS patients were more likely to undergo PCI from the femoral artery (14.1 vs. 9.9%, p0.019). The mean number of vessels treated (Table 2) was similar between the groups whilst the number of lesions treated (1.81±1.1 vs. 1.58±0.92, p = 0.003), total lesion length (32.1±21.7 vs. 26.1±17.6mm, p<0.001), the number of stents used (1.93±1.11 vs. 1.65±1.4, p-0.007), and total stent length (37.5±20.8 vs. 32.4±20.3, p<0.01) were greater for BA9-DCS patients. In 95.7% of procedures the patient would have been drug-eluting stent candidate i.e. a stent was placed that was <3.0mm in diameter or >15mm in length. Consistent with the patient demographics and higher bleeding risk, glycoprotein inhibitor use was less prevalent in the BA9-DCS cohort (4.5 vs. 8.6%, p = 0.012). Although there were more lesions successfully treated in BA9-DCS cohort, the number of residual lesions left at the end of the procedure were similar between the groups.

**Table 2:** Procedural data for patients treated with a BA9 drug-coated stent vs. PCI-DES population.

	DCS Cohort (n = 249)	PCI-DES cohort (n = 1630)	p value
Femoral access (%)	14.1	9.9	0.019
Chronic total occlusion PCI (%)	3.6	8.6	0.001
Left main PCI (%)	3.0	2.6	0.404
LAD PCI (%)	44.2	41.9	0.817
Left circumflex PCI (%)	23.3	20.6	0.218
Right PCI (%)	38.7	30.7	0.212
Saphenous graft PCI (%)	2.5	3.6	0.215
No. vessels treated (±SD)	1.31±0.52	1.29±0.52	0.360
No. lesions treated (±SD)	1.81±1.1	1.58±0.92	0.003
Total lesion length mm (±SD)	32.1±21.7	26.1±17.6	<0.001
Multi-lesion PCI (%)	48.1	35.3	0.004
Multi vessel (%)	19.6	17.9	0.435
Lesions >30mm (%)	37.7	26.2	<0.001
Thrombus extraction (%)	9.9	13.2	0.103
Distal protection used (%)	0.5	0.5	1.000
Atherectomy device (%)	8.9	5.6	0.129
Number stents used (±SD)	1.93±1.11	1.65±1.4	0.007
Longest stent mm (±SD)	27.7±10.0	24.1±8.2	<0.001
Mean stent diameter mm (±SD)	2.94±0.47	2.97±0.49	0.318
Total stent length mm (±SD)	37.49±20.8	32.4±20.3	<0.001
Bivalirudin use (%)	15.8	18.8	0.290
Glycoprotein inhibitor used (%)	4.5	8.6	0.012
No. lesions successful (±SD)	1.79±1.0	1.53±0.9	<0.001
No. residual lesions (±SD)	0.081±0.28	0.082±0.32	0.497

### Patient outcomes

**Fig 2:**
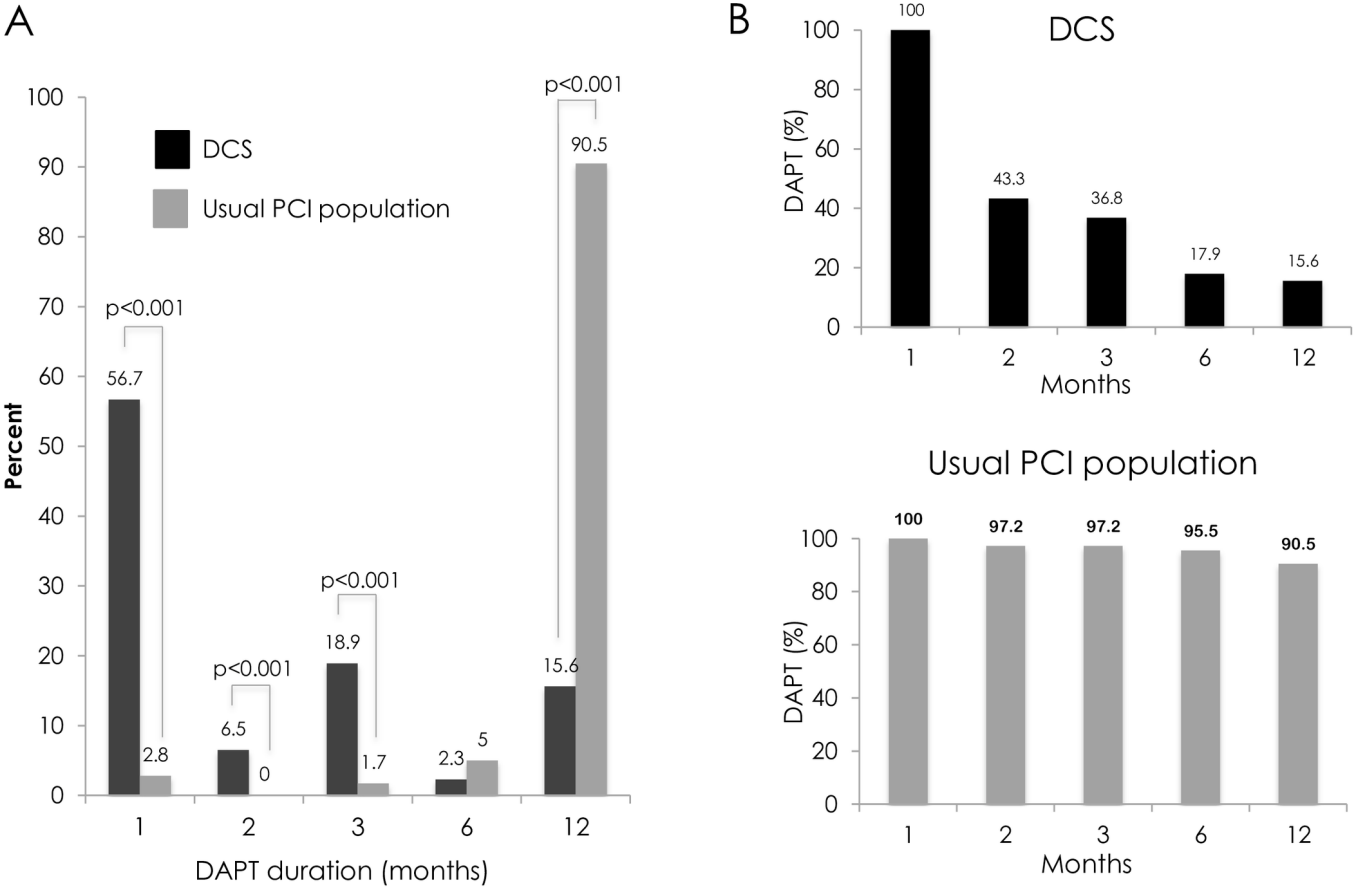
A) Dual anti-platelet duration (DAPT) in months by treatment group; B) Percentage of patients on DAPT at each time point by treatment group.

Physician-recommended DAPT duration was 3.3±3.9 months for BA9-DCS patients (Fig 2, panel A) and 11.3±2.4 months for PCI-DES patients (p<0.001). As a result, there were significant differences between the groups with respect to the proportions of patients on DAPT at all time points from PCI to 12-months (Fig 2, panel B). At mean follow up of 392±124 days, crude unadjusted stent-related event rates were low with definite or probable stent thrombosis occurring in 0.8% of the BA9-DCS cohort compared to 1.5% in the PCI-DES comparison (p = 0.64). One BA9-DCS thrombosis occurred on day 9 in a patient who received 3 BA9-DCS stents (all less than 2.75mm in diameter with a total stent length of 88mm) who was non-compliant with DAPT. The second BA9-DCS thrombosis occurred on day 25 during DAPT and was felt to be due to sub-optimal stent deployment. There were no definite or probable episodes of BA9-DCS thrombosis after the prescribed DAPT duration had been completed. There were two sudden unexplained deaths at day 114 in the BA9-DCS cohort that were categorised as possible stent thromboses.

**Fig 3:**
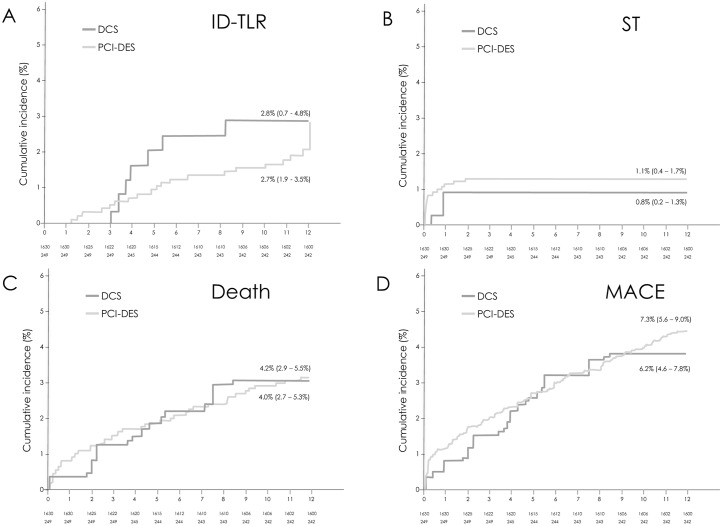
Adjusted Kaplan-Meier curves for ischaemia-driven TLR (ID-TLR, panel A), stent thrombosis (ST, panel B), death (panel C) and all MACE combined (panel D).

Crude unadjusted ischaemia-driven restenosis PCI occurred in 2.8% of the BA9-DCS cohort at a mean of 141.3±57.6 days compared to 3.4% in the PCI-DES cohort (p = 0.84). There was a numerical excess of unadjusted death in the BA9-DCS cohort compared to the PCI-DES population although this did not reach statistical significance (p = 0.26). However, adjusted clinical outcomes after propensity scoring were similar in both cohorts (Table 3) with no excess of stent thrombosis, ID-TLR or death in the BA9-DCS cohort. Overall MACE was also similar between the two cohorts. Adjusted Kaplan-Meier curves for ST, ID-TLR, death and all MACE are shown in Fig 3. BARC 3 or 5 bleeding occurred in 13 patients (5.3%) in the BA9-DCS cohort (at a mean of 134.9±141.8 days) with 55.6% occurring early whilst treated with DAPT. Myocardial infarction occurred in 4.8% (at a mean of 151.2±99.3 days) in the BA9-DCS cohort.

**Table 3:** Adjusted clinical outcomes after propensity scoring in patients treated with a BA9 drug-coated stent vs. PCI-DES population.

	DCS Cohort (n = 249)	PCI-DES cohort (n = 1630)	HR (95% CI)	p-value
Ischemia-driven TLR	2.8	2.7	1.05 (0.473:2.33)	0.905
Definite/probable ST	0.8	1.1	0.71 (0.26–1.91)	0.496
Death	4.0	4.2	0.96 (0.60–1.52)	0.850
All MACE	6.2	7.3	0.84 (0.59–1.21)	0.353

## Discussion

Our early clinical experience with a polymer-free BA9 drug-coated stent can be summarised as follows: 1) Patients in the BA9-DCS cohort were at high bleeding risk with advanced age, over-representation of female patients, low body weight, and a high prevalence of hypertension; 2) The BA9-DCS PCI procedures were complex with long lesions, frequent requirement for atherectomy and long lengths of stents implanted; 3) Clinician-determined DAPT duration in BA9-DCS patients was significantly shorted than the PCI-DES recommendation; 4) Despite longer lesions and abbreviated DAPT duration, the clinical outcomes were encouraging with low TLR and ST rates.

The identification of patients deemed to be at risk of bleeding with prolonged DAPT duration is an important component of PCI planning. Important risk factors for bleeding have been identified in several studies and include age, female sex, low body weight, chronic kidney disease and acute presentation [14]. Additionally, although many interventional operators use clinical judgement, there are several validated risk-scoring models to facilitate patient identification [15–16]. Most recently an analysis of the DAPT trial has provided further insights into balancing the risks of bleeding and ischaemia with longer duration of clopidogrel [17]. In the current study, the most frequent indication for Polymer-free BA9 drug-coated stent use was concurrent warfarin therapy. Data from the WOEST study reveal a striking 44.4% incidence of major bleeding with a 12-month course of triple therapy [18]. Therefore, it is understandable that operators wished to abbreviate DAPT duration in patients treated with warfarin. Current estimates of the numbers of patients undergoing PCI who are treated with chronic warfarin therapy range between 5–8% but with an aging population this frequency is highly likely to increase [19]. In this cohort with an indication for an abbreviated DAPT course there is a significant unmet need in current PCI practice.

One possible strategy to shorten DAPT therapy in patients at risk of bleeding is to implant a bare metal stent. Indeed, data from the run-in study to LEADERS-FREE suggests warfarin therapy was a frequent reason that interventional cardiologists reported for choosing a bare metal stent [20]. However, the current BA9-DCS data demonstrates complex disease in such patients with small vessels and long lengths of disease. Therefore, a bare metal stent driven strategy may well result in high rates of TLR, which itself has a significantly deleterious effect on patient outcomes. The Zeus study was one of the few studies that actively recruited patients at risk of bleeding, randomising participants to treatment with either bare metal stents or Endeavor stents. In the ZEUS trial, TLR was significantly higher in the BMS arm than the DES arm [21]. However as well as increased TLR, BMS implantation was associated with higher rates of myocardial infarction and stent thrombosis. Therefore, in many patients at risk of bleeding—despite concerns about long course of DAPT therapy—operators may opt to implant a drug eluting stent because of the prohibitively high risk of TLR with a bare metal stent strategy.

If a DES is implanted, operators are faced with a choice to either prescribe the recommended 12-month DAPT course (in patients with an ACS indication), or alternatively to abbreviate the course perhaps to 3 or 6-months (in keeping with emerging trial and registry data). However, persisting with a 12-month course places patients at significant risk of bleeding as illustrated by WOEST [18]. In the current study severe bleeding occurred in 5.3% of patients at 12-months follow-up, which whilst higher than in other studies is an acceptable frequency. However, it is important to note that over half of the significant bleeds occurred early after PCI while patients were still on DAPT. This illustrates the additive risk such patients are placed under when treated with long courses of DAPT. Additionally, it is now well established that bleeding after PCI is strongly associated with adverse patient outcomes. In the ACUITY study, a profound impact of protocol-defined major bleeding on mortality was seen with an observed OR of 7.55 at 30-days [9]. The negative impact of bleeding on intermediate-term outcomes may not only be restricted to larger bleeds. In a pooled analysis of GUSTO-IIb, PURSUIT and PARAGON, even minor bleeding had a significant impact on 6-month mortality [22].

Emerging data from recent observational studies of the randomised trial programs of the Endeavor and Xience stent provide support for the concept of a reduction in DAPT duration. However, as this is observational data derived from randomised trials, there are limitations in its applicability to everyday clinical practice. Such limitations include confounders such as treatment bias and also small numbers of patients receiving short DAPT duration [23,24]. Data is also accumulating from RCCT trials of shortened DAPT duration vs. standard duration with EXCELLENT, RESET, PRODIGY and OPTIMIZE all suggesting equivalency [25–27]. However, a recent meta-analysis of 11 such trials revealed a doubling of the frequency of stent thrombosis with short vs. long DAPT [28]. Importantly, mean lesion length was short and mean vessel size large in these trials limiting the applicability to everyday clinical practice. However, as result of these recent trials the most recent ESC guidelines on post-PCI anti-platelet treatment recommend a 6-months course of DAPT after DES implantation for stable angina, and as little as 3-months when patients are deemed to be at high risk of bleeding [10].

Given the lack of data in dealing with complex coronary disease in patients at high risk of bleeding, there is potential for new stent technologies to improve patient outcomes. The BioFreedom drug coated stent is a polymer-free platform resulting in rapid drug-elution profile that may allow shortened duration of DAPT without compromising TLR and MACE. In pre-clinical studies, superior strut coverage was demonstrated compared to bare-metal and sirolimus-eluting stents [29, 30]. In a first-in-man study, the polymer-free BA9 drug-coated stent demonstrated non-inferior late loss against the Taxus stent (0.17mm vs. 0.35mm, p<0.001), with similar frequency of MACE [11]. The recent LEADERS FREE trial studied the use of the BioFreedom stent in patients at high bleeding risk, and demonstrated reduced frequency of target lesion revascularisation and myocardial infarction in patients treated with a BA9-DCS stent compared to a bare-metal stent cohort [12]. In the current study we implanted the polymer-free BA9 drug-coated stent in a cohort of patients (the vast majority of whom were DES eligible) selected by the interventional cardiologist in whom it was deemed undesirable to commit the patient to a prolonged course of DAPT. Despite the rapid elution profile and thus the shortened DAPT, we did not observe any excess stent-related events such as ID-TLR or ST when compared to a PCI-DES population [31]. The findings of the present study whilst consistent with the results of the LEADERS-FREE study additionally suggest efficacy and safety the BA-A9 drug-coated stent in a real world population. Additionally, our study cohort was dominated by ACS patients (>80%) in contrast to the LEADERS-FREE study. This is of importance in assessing stent thrombosis give its rare occurrence in stable angina PCI. However, the study is underpowered to robustly define the stent thrombosis rates although these preliminary results are encouraging particularly given that the BA9-DCS cohort exhibited a high frequency of the risk factors associated with ST including ACS presentation, diabetes, longer lesions and longer stents compared to the PCI-DES population. Therefore, these preliminary data are hypothesis generating and provides the basis for future study.

The heterogeneity of DAPT duration observed reflects implanting physicians’ discretion, and interpretation of balancing the individual patient stent thrombosis and bleeding risks. This heterogeneity is likely a result of only early data being available regarding DCS use, and the absence of guidelines regarding optimal DCS DAPT duration. The 12-month recommendation in certain cases was due to the ACS presentation and due regard to the ACS guidelines. However, in these cases often the narrative was that 12-month was recommended due to an ACS presentation, but that the use of a BioFreedom facilitated DAPT discontinuation should a bleeding event or intolerance/allergy occur for example.

In considering the other limitations of this study, firstly—as this is observational non-randomised data—it is possible that selection and treatment biases confound the results. Secondly, as the patients were treated in only two centers the results of the polymer-free BA9 drug-coated stent in other centers might be different. Thirdly, as the numbers are modest we did not have the statistical power to analyse outcomes by variable DAPT duration. However, as this is observational data any conclusions from such an analysis are likely to be severely limited by treatment bias.

In conclusion, the outcomes from this preliminary study using polymer-free BA9 drug-coated stents in DES-type patients who are poor candidates for prolonged dual anti-platelet therapy are encouraging, with low repeat revascularisation and stent thrombosis rates. Further studies are warranted.

## Supporting Information

S1 File DES and BioFreedom for propensity matching PLOS (basic unadjusted data). (XLSX)

S2 File Data file DCS for PLOS (data prepared for propensity scoring). (XLSX)

S3 File DCS-DES report (results of propensity scoring). (DOCX)
