# Supplementary material for: Early Clinical Experience with a Polymer-Free Biolimus A9 Drug-Coated Stent in DES-Type Patients Who Are Poor Candidates for Prolonged Dual Anti-Platelet Therapy
Source: PLoS One. 2016 Jun 30;11(6):e0157812. doi: 10.1371/journal.pone.0157812 (PMC4928951; doi:10.1371/journal.pone.0157812)

Contents

[Unadjusted analysis of event rates 2](#_Toc447626059)

[Adjusted analysis of event rates 2](#_Toc447626060)

[Unadjusted Kaplan-Meier curves 4](#_Toc447626061)

[Adjusted Kaplan-Meier curves 8](#_Toc447626062)

## Unadjusted analysis of event rates

Table 1 displays event rates (Kaplan-Meier estimates) for the 249 DCS and 1630 DES patients at 1 years. In order to compare the DCS versus DES in terms of event rates, a Cox proportional hazard model was fitted to the data. Patients not experiencing an event were censored either at the time they withdrew from the study if they withdrew early or at the latest after 360 days if they completed the study. The hazard ratio needs to be understood in the following way: if it is higher than one then it means the risk of experiencing an event in the DCS is higher than the risk in the DES group.

**Table 1: Unadjusted event rates**

| Parameter | Statistics | DCS stent (N=249) | DES stent (N=1630) | Hazard ratio | P-value |
| --- | --- | --- | --- | --- | --- |
| All death | N(%) | 13 (5.22%) | 61 (3.74%) | 1.407 (0.773:2.56) | 0.2621 |
| Definite or probable stent thrombosis | N(%) | 2 (0.8%) | 17 (1.04%) | 0.768 (0.178:3.325) | 0.7235 |
| Clinically-driven target lesion revascularization | N(%) | 7 (2.81%) | 44 (2.7%) | 1.05 (0.473:2.33) | 0.9053 |
| Composite of death or definite/probable ST or clinically-driven cTLR | N(%) | 21 (8.43%) | 111 (6.81%) | 1.253 (0.786:1.998) | 0.3420 |

## Adjusted analysis of event rates

We also performed a propensity score analysis in order to balance for important covariates that might bias estimates for causal inferences. The possibility of bias arises because the apparent difference between these two groups of patients may depend on characteristics that affected the decision whether or not a patient received a given stent instead of due to the effect of the stent per se. Propensity score methods will try to adjust the analysis by taking into account the characteristics of a patients that might have influenced his or her assignment to a particular stent group. Below is the set of variables that we controlled for in this report:

1. Age
2. Sex
3. Weight
4. Acute coronary syndrome
5. Cardiogenic shock pre-procedure
6. Previous MI
7. Angina status
8. Diabetes status
9. History of renal insufficiency
10. Congestive heart failure
11. Q-wave on ECG
12. hypertension
13. CVA
14. PVD

A propensity score for each patient was obtained from a logistic regression for the group variable predicted from the variables above. Here we used a method called Inverse Probability of Treatment Weights (IPTW). More precisely, one estimates the probability that a particular patient is assigned to one of the two groups as a function of that individual's covariates (the propensity score). Each individual observation is then given a weight equal to the inverse of this propensity score to create two pseudo-populations of exposed and unexposed patients who now represent what would have happened to the entire population under those two "treatment" conditions. The advantage of this method is that it is inclusive as it uses all patients in a study, therefore no loss of sample occurs as in other conditioning methods such as matching or stratification. We also normalized the weights by dividing them by the mean weight.

Table 2 reports the adjusted event rates (adjusted Kaplan-Meier estimates) and the adjusted hazard ratios and p-value. There is no difference between the two analyses. Looking at the hazard ratios and p-values, the adjusted analysis tends to improve the outcomes in the DCS group compared to the DES group.

**Table 2: Adjusted event rates**

| Parameter | Statistics | DCS stent (N=249) | DES stent (N=1630) | Hazard ratio | P-value |
| --- | --- | --- | --- | --- | --- |
| All death | N(%) | 4.0% | 4.2% | 0.956 (0.602:1.519) | 0.8502 |
| Definite or probable stent thrombosis | N(%) | 0.8% | 1.1% | 0.707 (0.261:1.914) | 0.4956 |
| Clinically-driven target lesion revascularization | N(%) | 1.7% | 2.9% | 0.58 (0.303:1.11) | 0.1000 |
| Composite of death or definite/probable ST or clinically-driven cTLR | N(%) | 6.2% | 7.3% | 0.842 (0.587:1.21) | 0.3529 |

## Unadjusted Kaplan-Meier curves

This section shows unadjusted KM curves for the different events definition along with the KM estimates and numbers at risk. By unadjusted, we mean that the propensity scores analysis is not used.

**Death**


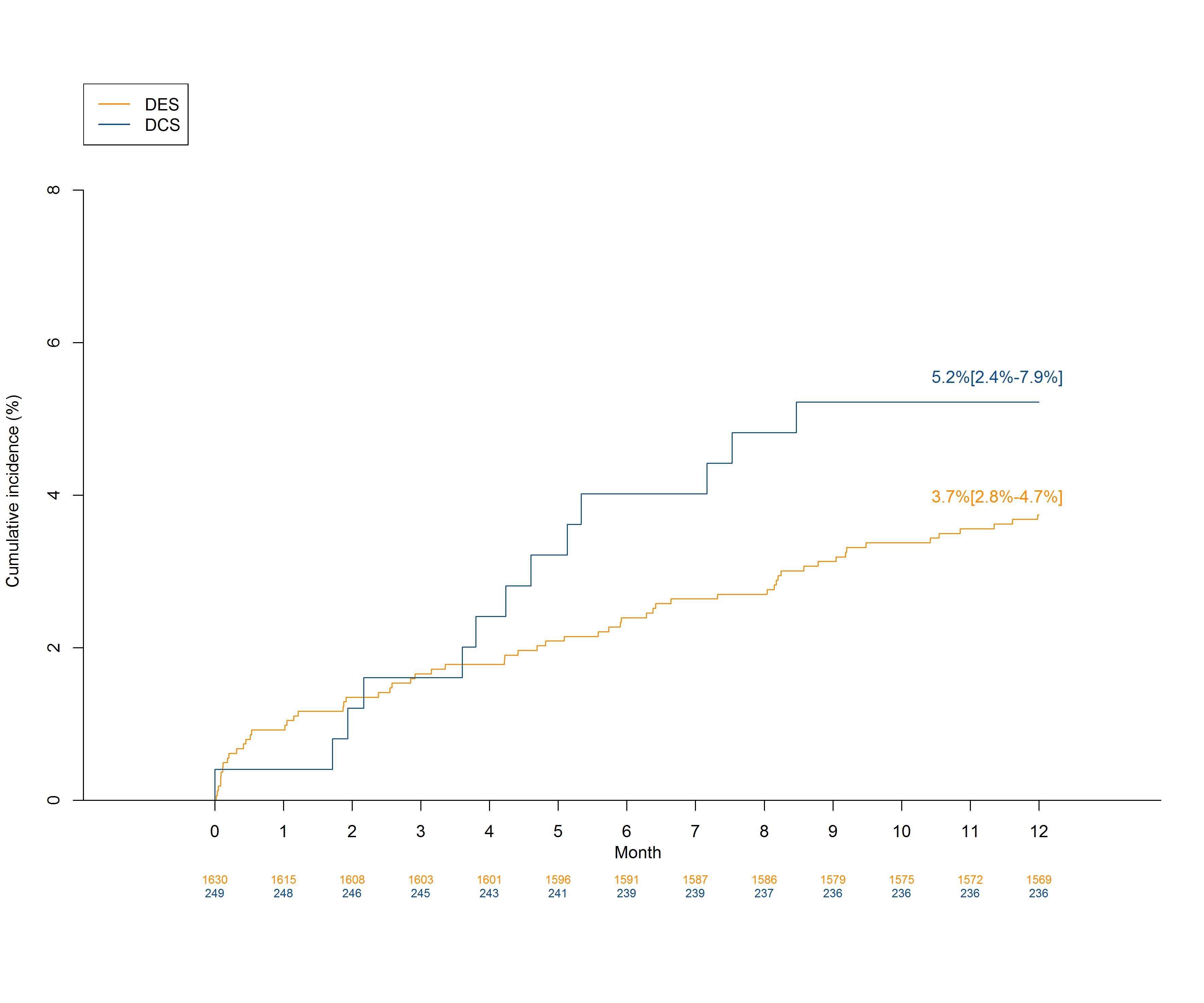


**Clinically-driven TLR**

**
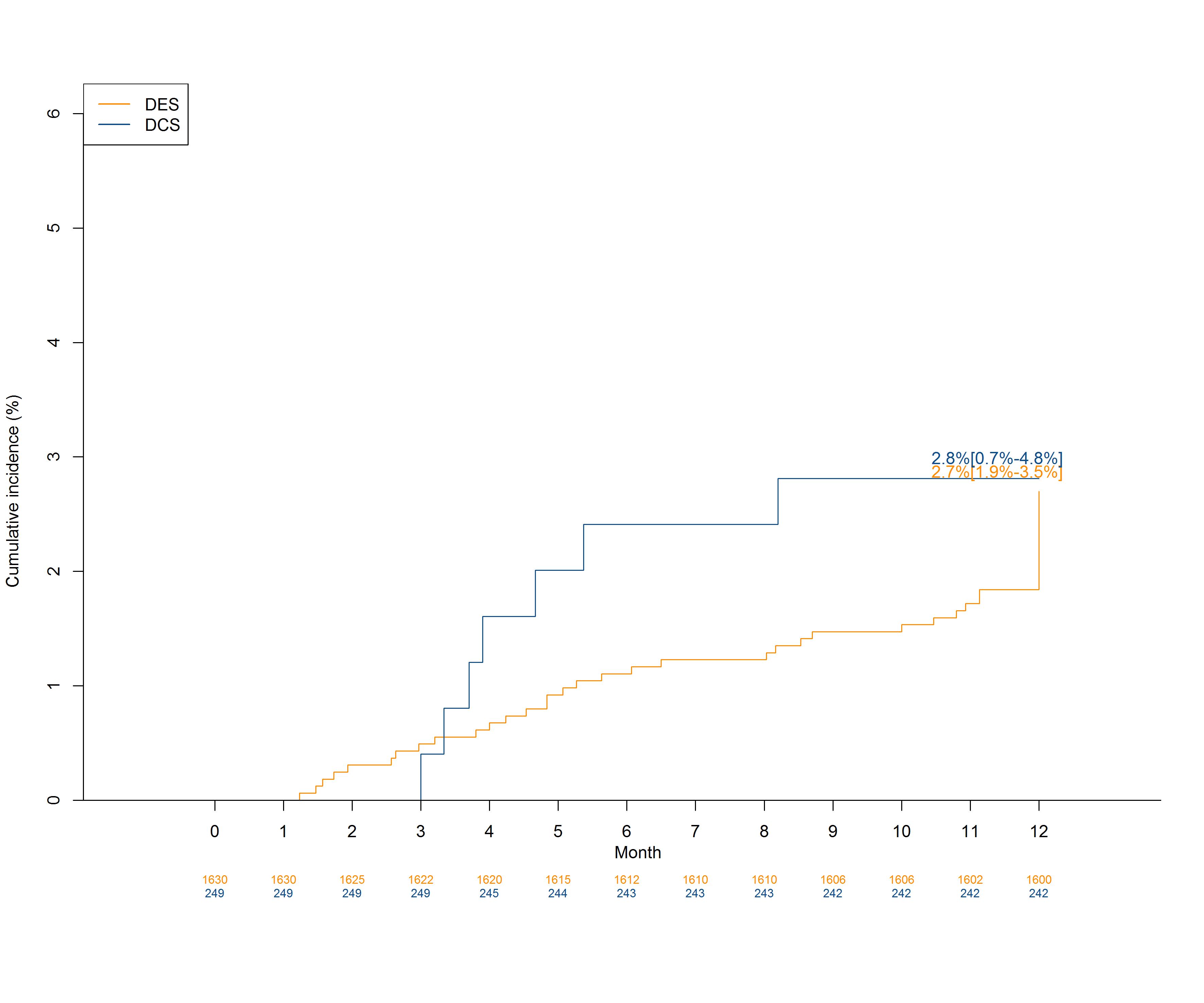
**

**Definite/probable ST**


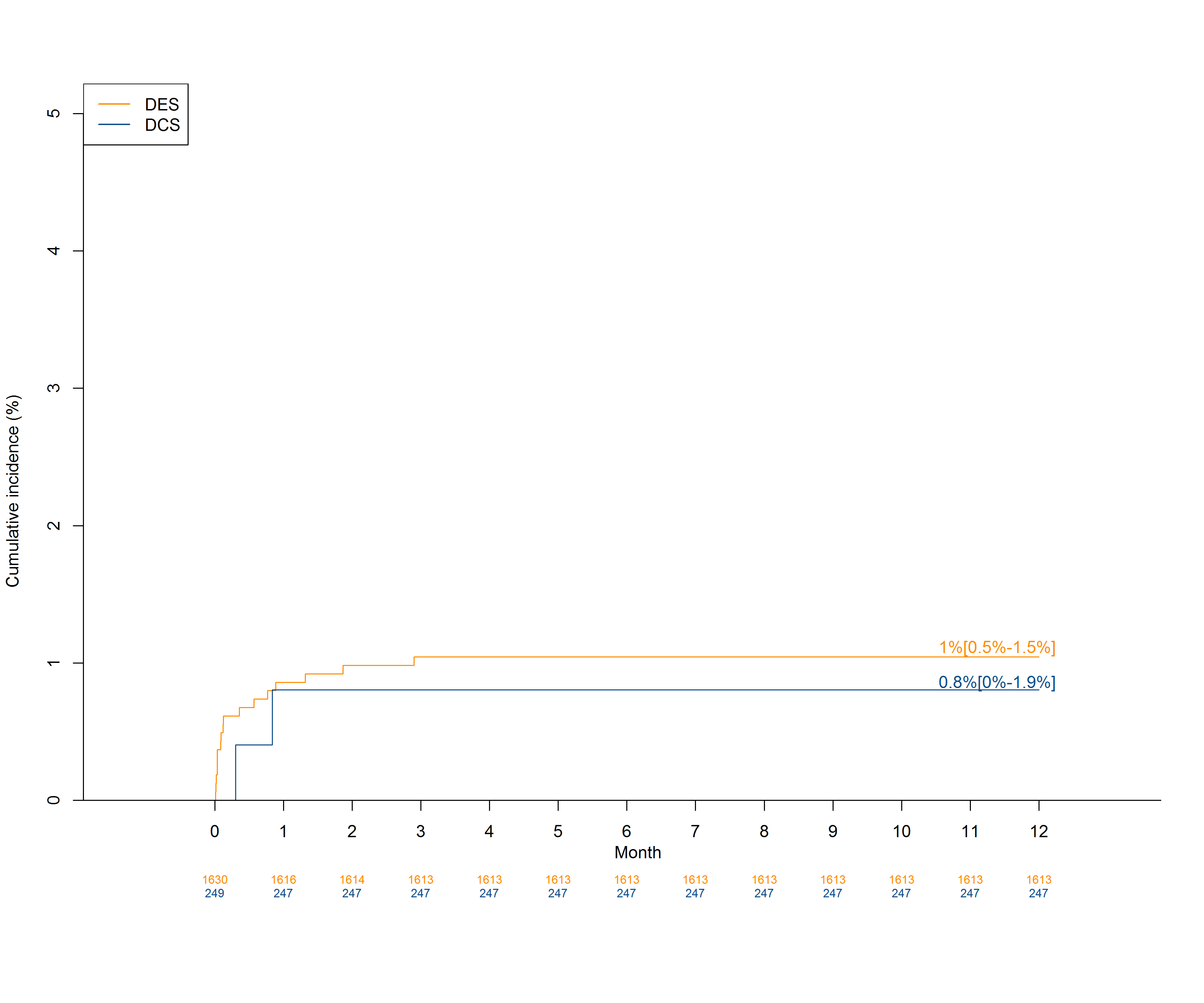


**Composite of death, ci-TLR or definite/probable ST**


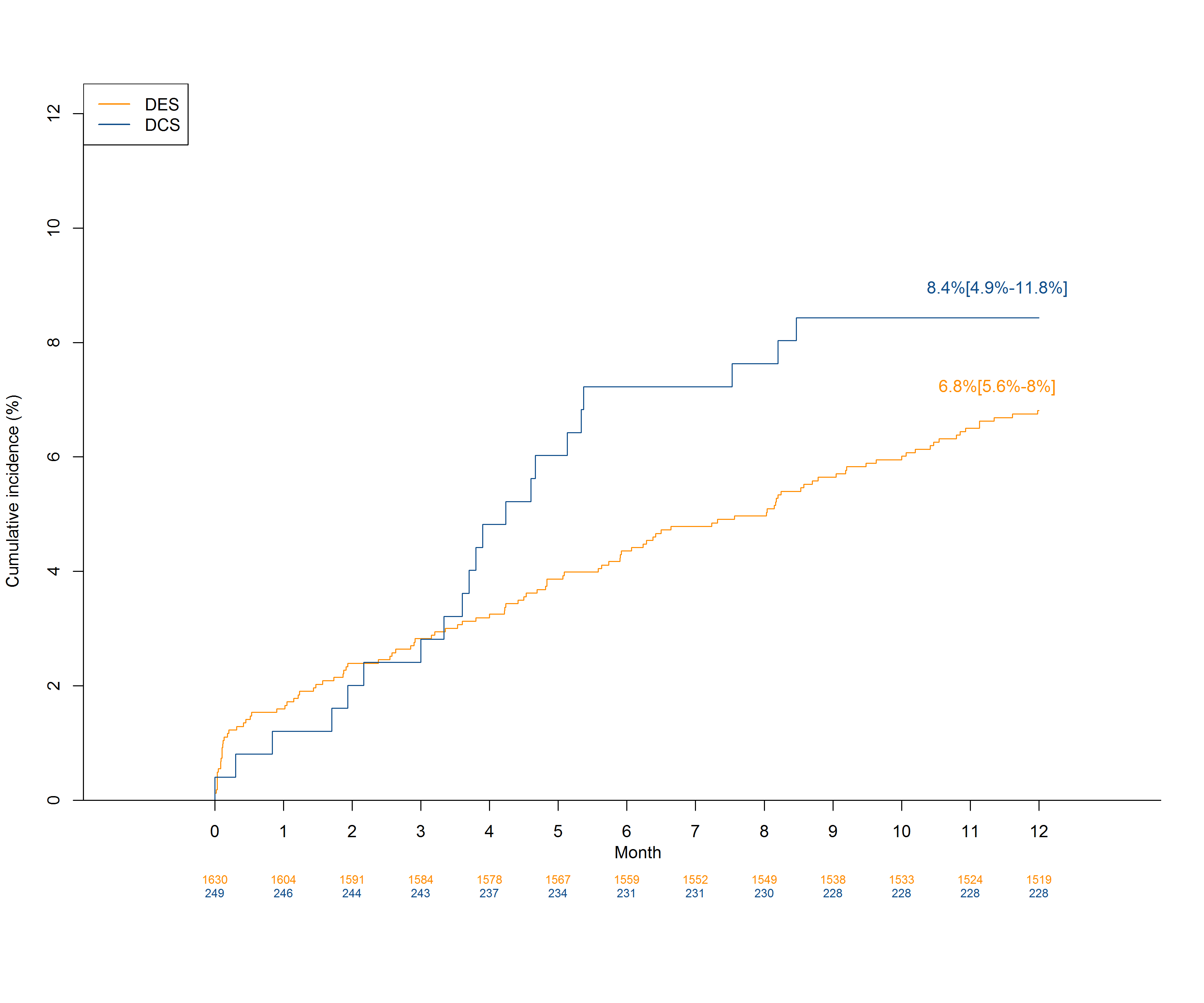


## Adjusted Kaplan-Meier curves

This section shows adjusted KM curves for the different events definition along with the KM estimates and numbers at risk. By adjusted, we mean that each patient is weighted according to the weight determined by the inverse of their propensity score. Those KM curves can be interpreted as being “adjusted” for the baseline variables described in above.

**Death**

**
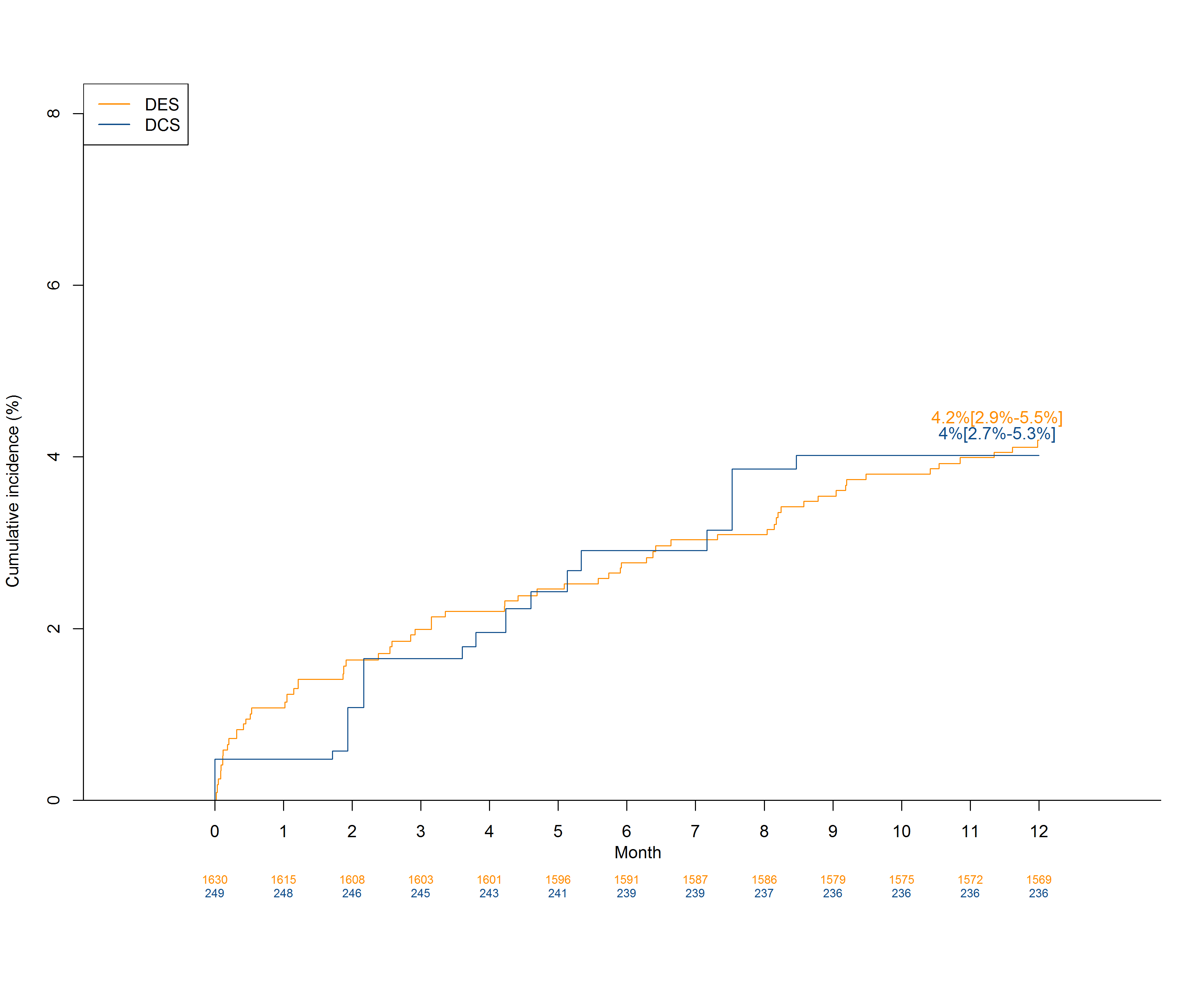
**

**Clinically-driven TLR**

**
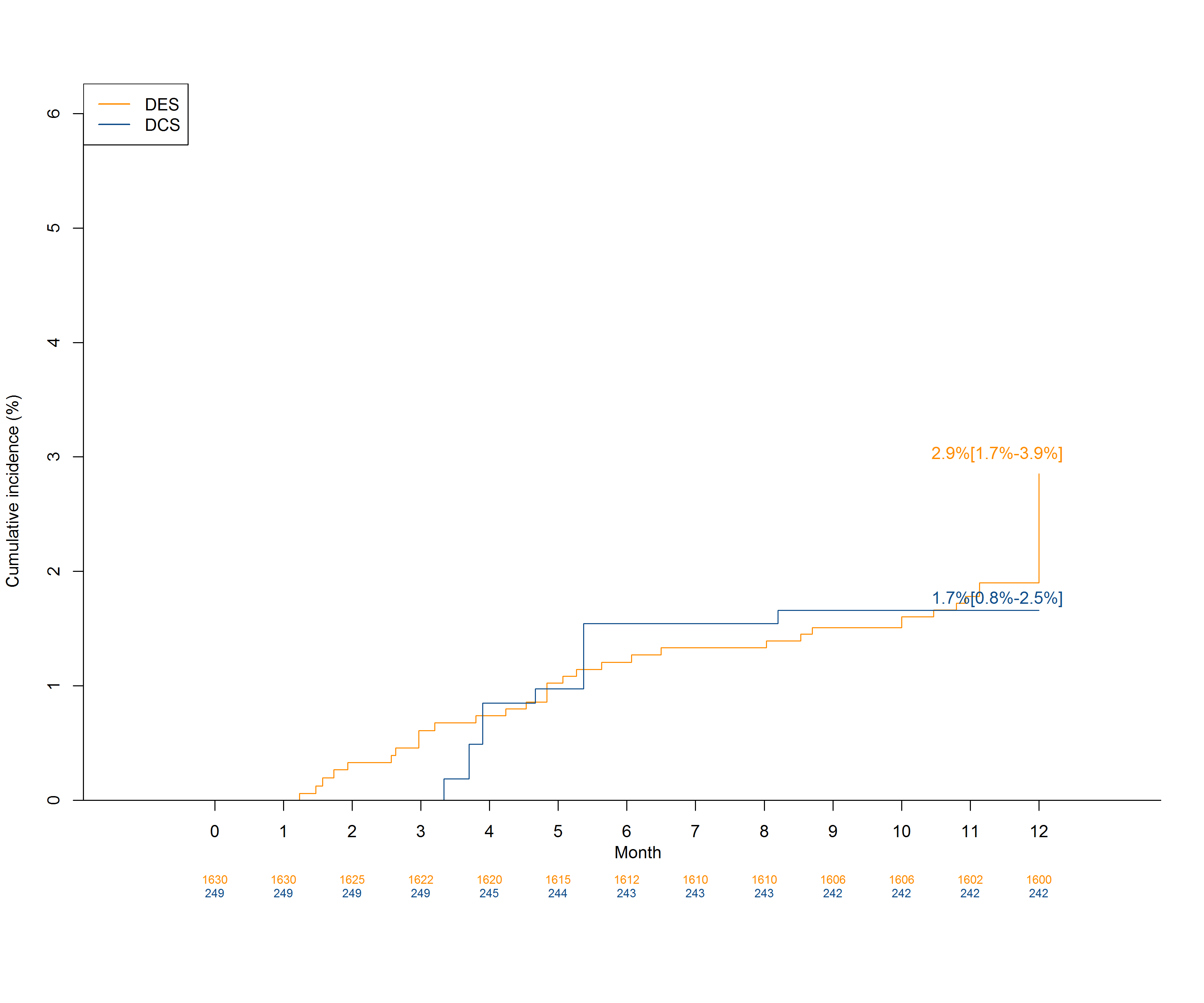
**

**Definite/probable ST**


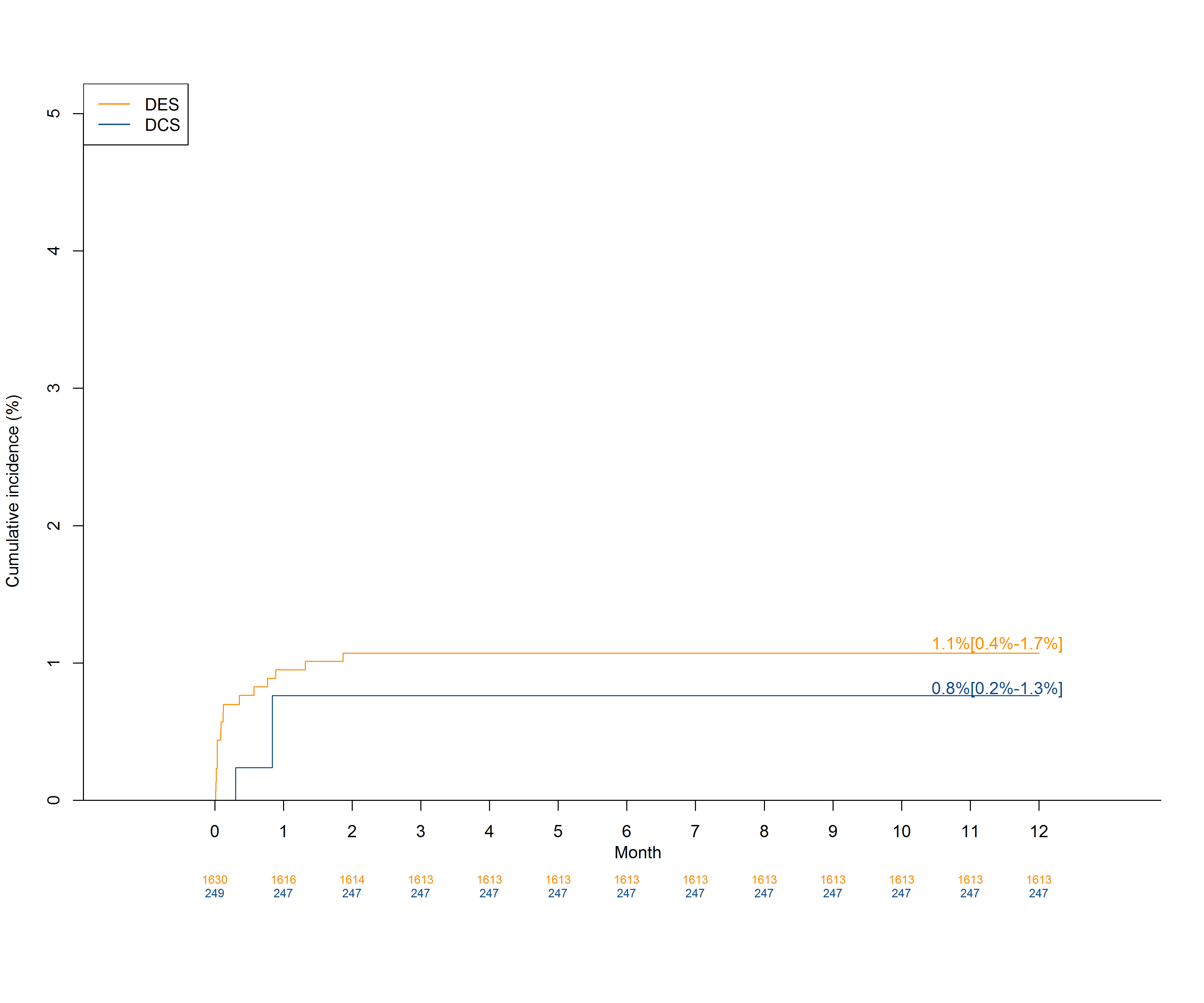


**Composite of death, ci-TLR or definite/probable ST**


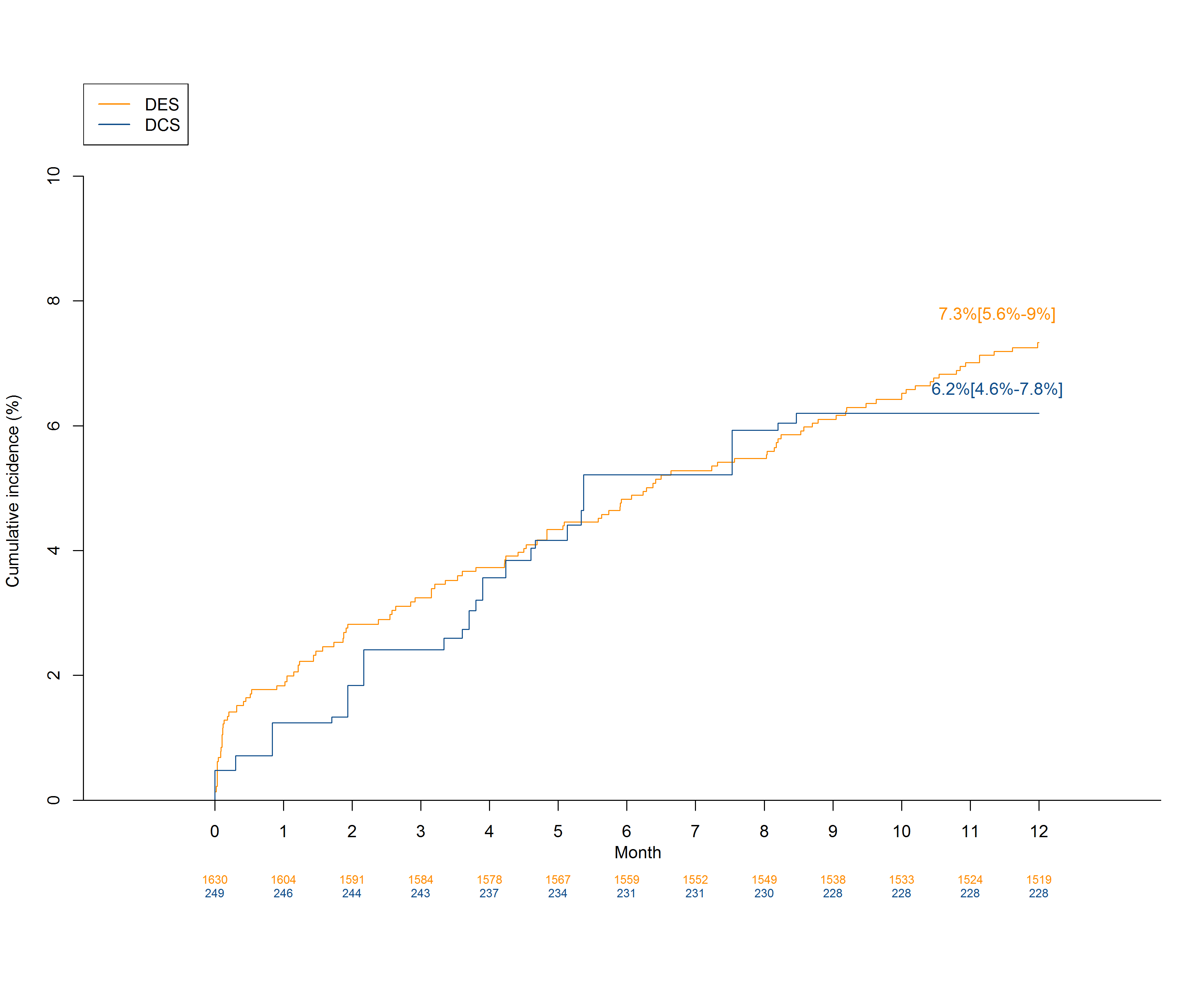

Supplement: S3 File — (DOCX) [file pone.0157812.s003.docx]
